# Topics of Public Interest

**Published:** 1905-10

**Authors:** 


### TOPICS OF PUBLIC INTEREST.

### Pneumonia Commission.

THE Medical Commission for the Investigation of Acute Respiratory Diseases, which was formed at the request of the Department of Health of the city of New York, and begun its work in October, 1904, has brought together for publication a number of reports on the special studies already made under its general direction. These papers will appear in the forthcoming issue of the *Journal of Experimental Medicine,* published under the auspices of the Rockefeller Institute for Medical Research, a majority of whose directors are among the members of the commission, advance sheets of which are furnished for comment.

This commission was constituted as follows: Dr. Edward G. Janeway, of New York, president; Dr. William Osler, of Baltimore, vice-president; Dr. T. Mitchell Prudden, of New York, secretary; Dr. Theobald Smith, of Boston; Dr. William H. Welch, of Baltimore; Dr. Frank Billings, of Chicago; Dr. John H. Musser, of Philadelphia; Dr. L. Emmett Holt, of New York; and Dr. Francis P. Kinnicutt, of New York, in association with Dr. Thomas Darlington, president, and Dr. Hermann M. Biggs, general medical officer, of the Department of Health.

Drs. Darlington and Biggs sign a preface to the reports, which outlines as follows the causes and objects of the investigation:

For some years the problem presented by the very large and constantly increasing deathrate from the acute respiratory diseases has been the cause of serious concern to the Board of Health of New York city, and in August, 1904, with the consent of the mayor and the Board of Estimate, it appointed a medical commission to conduct an investigation as to the causes for the great prevalence of the acute respiratory diseases in the city, with the hope that some means could be devised for reducing the excessive morbidity and mortality from this cause.

The problem has long been recognised as one of the first in sanitary importance, and efforts have frequently been made to find some method for its solution. The inherent difficulties of the problem, however, are so great and the other questions pressing for consideration have been so numerous and important that no determined efforts have hitherto been made to discover means or devise methods to effectually meet the situation.

Succinctly stated, the situation is this: During the last 20 years, the general deathrate in New York city has fallen 25 per cent, and the deathrates from all the principal causes of death have fallen from 10 per cent. to 35 per cent., excepting in four groups of diseases, in which there has been an increase. These are: (1) the acute respiratory diseases; (2) cancer; (3) the diseases of the heart and blood-vessels; (4) the diseases of the kidney. The increases in these groups have been from 10 per cent. to 30 per cent., or 35 per cent.; in the acute respiratory, being from 10 per cent. to 15 per cent. In the first half of 1904, there was an extraordinary increase in this group, the deaths from the acute respiratory diseases alone forming no less than 23 per cent. of the total death rate.

The importance of the situation, so far as this group is concerned, is more forcibly brought out when we compare the percentage which the total deaths from the acute respiratory diseases bear to the total deaths from all causes. In 1883, it was between 7 per cent. and 8 per cent., and in 1893, nearly 16 per cent., showing a relative increase during this period of 100 per cent.

A consideration of the etiology of the acute respiratory diseases, brings out even more strongly the sanitary importance of the problem. There can now be no question that the exciting cause in each one of the diseases of this group is a microörganism, which requires such conditions for its growth and multiplication as are usually found only in the living body. These organisms do not, under natural conditions, multiply to any extent outside of the tissues or cavities of the body. The infection, when it occurs, must practically always be the result of communication, directly or indirectly, from one human being to another. The conclusion, therefore, seems justifiable, that these diseases are essentially communicable, and, however great the inherent difficulties of the problem may be, theoretically at least, they should be, to a greater or less extent, preventable.

The difficulties in the way of prevention arise largely from the wide distribution of the organisms. Streptococci, pneumococci, influenza, bacilli, singly or combined, are present in almost all morbid conditions in the respiratory tracts of the inhabitants of large cities, and probably also to a very large extent in the respiratory tracts of healthy individuals. The situation is much like that existing with relation to diphtheria, excepting that these organisms probably live more readily and for longer periods of time in the body cavities of healthy individuals than do the diphtheria bacilli, and the latter perhaps have (at least in children) greater pathogenic activity.

The Board of Health, looking forward to the study of this problem in the revision of the Sanitary Code made in 1903, included this group of diseases in the class in which partly voluntary and partly compulsory notification was required. This provision of the Sanitary Code, however, has not yet been enforced. It is believed now that provision should be made for this.

It has always been the feeling of the medical officers of the board that no really important effective measure could be taken in relation to any preventable disease unless the sanitary authorities first had some fairly comprehensive information as to the prevalence and distribution of the disease—such information as can only be gathered from the systematic notification of cases.

As the problem under consideration is not simply one which concerns New York, but one which almost equally concerns all the large cities of the United States, it was felt to be important that the commission should have a distinctly representative character, in the hope that the influence which it would exercise would be broader and more effective. As it seemed desirable to the commission to carry on investigations in other laboratories, in other cities, suitable arrangements were made for defraying the expenses of these investigations by the Department of Health of New York City. The facilities of the laboratories of the Department of Health were also placed at the command of the commission for assisting in various lines of investigation in New York.

In an "introductory note to studies on the pneumococcus under the auspices of the medical commission," Dr. Biggs makes the following statements:

It was decided to concentrate attention at first upon lobar pneumonia in both its bacteriologic and its clinical aspects . Among the studies upon the bacterial excitant of lobar pneumonia which it was deemed wise to pursue were the following:

(1) A study of the occurrence and virulence of pneumococcus and organisms related to or resembling this, in the human mouth in health and disease; (2) the evidence of variations in virulence of the pneumococcus; (3) the occurrence of the pneumococcus in children's hospitals, homes, and asylums, with a study of the bacteria of mouths before and after outbreaks of pneumonia; (4) the vitality of the pneumococcus under various conditions; (5) a study of mouth disinfection.

It was the sense of the commission that one of the earliest phases of research, upon which much of the subsequent work would depend, was the determination, for the identification of species, of the characters of the pneumococcus, this investigation to include a comparative study of the coccus from cases of true lobar pneumonia and of that from normal mouths and throats hitherto generally assumed to be identical with the pneumococcus; also, to include such a study of streptococci as will suffice for their separation from the pneumococcus.

The method adopted in these studies was (1) to secure the coöperation of bacteriologists in various places, who should make independent studies along the lines suggested; and (2) to establish a central laboratory or "clearing house," to which ultimately cultures from various independent workers should be sent for comparative study under a single observer. It was hoped that by the establishment of this central laboratory with its large number of available cultures, light might be thrown upon atypic forms, variations, etc., and greater precision achieved in determining the characters relied upon for identification.

The commission secured the coöperation, in making the special studies referred to, of Dr. W. T. Longcope, of the Ayer Clinical Laboratory in Philadelphia; of Drs. C. W. Duval and Paul Lewis, of the city Hospital in Boston; of Dr. William H. Park and his associates in the laboratories of the Department of Health of New York City; of Dr. Leo Buerger, of Mt. Sinai Hospital, New York; of Professor F. C. Wood, of the College of Physicians and Surgeons, New York; and Dr. Chas. Norris, of New York.

The coöperation of Prof. Philip Hanson Hiss, of the College of Physicians and Surgeons, New York, was secured in the organisation and direction of the work of the central laboratory at the College of Physicians and Surgeons.

While various other lines of study are under way, it has been thought wise to publish at this time together the several independent studies which follow, in the belief that they may be useful not only in the further work of the commission, but of others engaged in similar lines of research.

The investigators' reports of the details of their laboratory work, along their several lines, are, as might be expected, extremely technical. Many, even, of the announcements of conclusions reached are so purely scientific, in both interest and phraseology, as to be out of place in a lay journal. Some of the conclusions, however, are of general interest, although separated from their context.

Drs. William H. Park and A. W. Williams report on a study of pneumococci in the research laboratory of the Health Department, where various experiments were made in the inoculation of rabbits, white mice, and sheep with pneumococcus cultures. Among their conclusions are the following:

Typic pneumococci were present during the winter months in the throat secretions of a large percentage of healthy individuals in city and country. . . .

Since the virulence of pneumococci may be rapidly increased for a susceptible species of experimental animal by successive passage, and since pneumococci obtained from most pneumonias are more virulent for experimental animals than are those obtained from healthy individuals, therefore the virulence of pneumococci from cases of human infection is probably increased for human beings; hence cases of pneumonia should be considered to a certain degree as contagious and, since the virulence of the pneumoccocus may be quickly increased and since the organism is very prevalent in normal sputum, all possible measures should be taken to restrict public expectoration.

In regard to some of the experiments at the Ayer Clinical Laboratory in Philadelphia, Drs. Warfield T. Longcope and W. W. Fox say that "the results suggest that during the winter months the pneumococcus has a wide distribution, and that at this time a large percentage of healthy individuals harbor virulent pneumococci in their buccal cavities. These months precede those in which pneumonia is most prevalent. It is almost certain that some persons always have virulent pneumococci in their saliva.

From the laboratories of Bellevue and allied hospitals comes a report by Drs. Charles Norris and Alwin M. Pappenheimer on "a study of pneumococci and allied organisms in human mouths and lungs after death." They say:

It follows logically, from the results obtained in this experiment, that the cultural findings after death are no guide to the bacterial contents of the lungs during life, and that any deductions made from such findings are unreliable and deceptive. Granted that our explanation be correct, there is every reason to believe that any of the microörganisms present in the mouth and pharynx, and in many cases in the stomach contents, may enter the lungs, and, if the conditions be suitable, increase in numbers, during the time between death and the examination of the lungs. . . .

Our studies have thrown no light whatever upon the conditions which determine the onset of lobar pneumonia in apparently healthy persons. Moreover, we have been unable to draw conclusions as to the presence of pneumococci in the lungs during life, or as to the channels by which they gain access thereto.

Following are some of the conclusions of Professor Philip Hanson Hiss:

It seems, therefore, more than probable that practically every individual, at least during the winter season, when exposed to environmental conditions such as those existing in New York City, acts as the host at some time or other, and probably at repeated intervals, of organisms of the true pneumococcus type.

None of the supposedly normal individuals examined by us had had a recognised pneumococcus infection or "cold" within a recent time, nor, with two well-marked exceptions, did any symptoms of infections develop in those whose mouths were found to contain pneumococci.

These two exceptions, detailed in an earlier section, strongly suggest the etiologic relations of pneumococci to some, at least, of the "common colds."

Prof. Francis Carter Wood, of the College of Physicians and Surgeons, closes an elaborate study with the following statements:

The conclusions of practical importance which can be drawn from the facts given in this paper are as follows:

1. The life of the pneumococcus in moist sputum is of considerable duration, the average period being less than two weeks, unless the material is exposed to direct sunlight. But as such sputum does not give off bacteria even when exposed to strong currents of air, it may be considered as innocuous, except to persons handling clothes, bedding, etc., which have recently been contaminated. Under ordinary conditions, however, this sputum dries in the course of a few hours or days. The dried masses retain their virulence for a long time, and if deposited on the floor or on the bedding of the patient, may be powdered mechanically, and sweeping, dusting or brushing the contaminated articles, will distribute pneumococci in the air. Fortunately, however, the organisms in the sputum do not remain long in suspension, and die off rapidly under the action of light and desiccation. In sunlight or diffuse daylight the bacteria in such powder die within an hour, and in about four hours if kept in the dark. The danger of infection from powdered sputum may, therefore, be avoided by ample illumination and ventilation of may, therefore, be avoided by ample illumination and ventilation of the sick room, in order to destroy or dilute the bacteria, and by the avoidance of dry sweeping or dusting. Articles which may be contaminated and which cannot be cleaned by cloths dampened in a suitable disinfectant, should be removed from the patient's vicinity.

2. When a person suffering from a pneumococcus infection coughs, sneezes, expectorates, or talks, particles of sputum or saliva are expelled from the mouth which may contain virulent pneumococci. Such particles remain suspended in the air for a number of hours if the ventilation of the room is good. They may be inhaled by persons in the vicinity of the patient, or they may be deposited upon various articles in the room. Whether suspended in the air or dried on surrounding objects, the writer's studies show that they become harmless in a very short time, about an hour and a half being the extreme limit, while many of the pneumococci in the spray perish in a few minutes, especially if exposed to strong light.

In the light of these experiments, the risk of infection from the pneumococcus is largely confined to those in direct contact with the person whose excreta contains the organism.—*American Medicine.*

---

### Improved Methods of the Twentieth Century Doctor.

**Much of the Credit Due to Americans. The Growth of Specialists and of a Liberal Spirit.**

[*New York Tribune*, September 24, 1905]

HE ENTERS your chamber quietly, slowly, and he smiles when you turn on your pillow and look at him. He asks you a few mild mannered questions and holds your hand. He gently pushes under your tongue a glass tube, and then nods to himself as if hailing as an old friend some thought passing through his mind.

"Just as I imagined," he says. "Been eating too much. Your stomach does not want medicine, but a vacation. You want to slow down a bit. Take more time for your meals, but eat less. Chew as well as swallow."

And to prompt your memory, he writes down a few words on a prescription blank. As quietly as he came he has gone. This is the twentieth century doctor.

The terrors which were once associated with the physician and surgeon are fast disappearing. A century ago one would have awaited the coming of his doctor only with apprehension. Where the modern practitioner would simply advise rest and an abstemious diet, his predecessor would have bled the patient after making him swallow some vile tasting compound, whose virtue was believe to be in proportion to its repulsiveness. He would have opened a vein in the wrist, or he would have applied leeches to drink the blood.

For much the same reasons that the doctor has become gentler toward his patient, he has grown more liberal toward his fellow practitioners. Different schools of medicine do not fight one another with the same animosity that once characterised them. Although the "regular" physician still hates the quack and charlatan as he did in the days of Galen, nevertheless he is far more friendly with a brother practitioner who has been graduated at a different school than his own and who employs different methods in his treatment of disease.

A striking illustration of the more fraternal spirit of the twentieth century doctor is shown just at the present time by the proposed union of the two great rival medical societies of this state. After more than twenty years of warfare, they may at last decide to lay down their arms and march under a common flag. Committees representing the New York Medical Association and the Medical Society of the State of New York are now conferring together, and the announcement of the formal amalgamation of the two organisations is expected in the near future. An act was passed by the last state legislature, which enables the two organisations to petition the supreme court together to permit them to consolidate under the name of the older body,—the Medical Society of the State of New York. The petitions are now being prepared for presentation.

The split was caused by a difference in opinion among the allopaths as to their attitude toward the homeopaths and other new schools of medicine. One faction held that a "regular" physician was at liberty to hold consultations with any legally licensed practitioner, whether he be an allopath or a homeopath. The other faction argued contrarywise. The liberals formed the medical society, while the conservatives were organised as the medical association.

Even a casual contrast of the past and present methods of the New York doctor in treating his patients shows how much less the world suffers now than in the "good old days." For example, the lunatics of Manhattan island one hundred years ago were compelled to submit to the "tranquilliser" or the "gyrator." When they grew lively they were treated to the "tranquilliser," a sort of straitjacket, in which they were strapped hand and foot. Should they become drowsy, they were placed in another machine, known as the "gyrator," and were whirled about like dancing dervishes.

In those days surgeons operated without the use of ether. America had not yet contributed anesthesia to the world, and thus opened a way through "the realms of painless oblivion" for the bold use of the knife. Patients who now fall asleep upon the operating table and awaken to find their broken bones set or ruptured organs in place cannot realise the agony of their forefathers, who were conscious of every move of the surgeon's hand. A medical man who had to undergo an amputation before the days of anesthesia tells of his experience, as related by Dr. James Gregory Mumford, of Boston, in "A Narrative of Medicine in America," as follows:

"During the operation, in spite of the pain, my senses were preternaturally acute. I watched all that the surgeon did with a fascinated intensity. I still recall with unwelcome vividness the spreading out of the instruments, the twisting of the tourniquet, the first incision, the fingering of the sawed bone, the sponge pressed on the flap, the tying of the blood vessels, the stitching of the skin and the dismembered limb lying on the floor."

Some of the early machines which were used for the replacement of bones caused the patient almost as much agony as the mediæval rack. "The occasional use of pulleys," says Dr. Frank P. Foster, editor of "The New-York Medical Journal," "is well remembered by some of us who are still living, to say nothing of the booted foot in the armpit. . . . The old methods of extension of the limb were painful, required protracted confinement in bed, and often produced ulceration of the parts on which the extending noose pressed."

Prior to 1652 the barbers of New York were classed as surgeons, and performed operations which were more painful than skilful. It appears that the surgeons of that time were especially jealous of the barbers, and succeeded at last in securing an act which prohibited the tonsorial artists from interfering with their practice.

Americans have had a foremost part in disarming surgery and medicine of its old terrors. Dr. Reid, of Rochester; Dr. Bigelow, of Boston, and various other American surgeons substituted manipulation instead of the old nerve-racking pulleys for the replacement of bones. Dr. Gordon Buck, of this city, invented an apparatus to extend a limb while the broken parts of a bone are setting, and thus prevent deformity or the shortening of the leg or arm. This devise did away with the ulcerating noose. Dr. James L. Little, a New-York surgeon, went still further in adding to the comfort of the patient by his use of the plaster of paris splint. By means of this splint a patient is now able to go about on crutches a few days after an accident, instead of languishing for weeks in bed.

As Dr. Foster points out in "The Medical Journal," surgery now does not wait until the swelling which follows setting the bones subsides before using the plaster cast. Dr. Henry B. Sands, another New-York practitioner, "demonstrated that it was unnecessary to wait even for the swelling to occur. He made the necessary extension at once, with the patient anæsthetised, and applied the plaster cast immediately. He also showed that not only fractures below the knee, but almost all fractures of the limbs as well, could advantageously be treated in this way."

Among other New York men who have succeeded in alleviating pain and deformity by more skilful yet less painful methods is Dr. Lewis A. Sayre, who straightened hunchbacks and enabled children stricken with hip disease to go skipping about the streets. Pott's disease, Dr. Sayre declared, was curable in its earlier stages, and therefore if this tuberculous disease of the spine be attacked when the patient is young, his deformity might be cured. Dr. Sayre treated the patient by suspending his head by means of a tripod, so that the weight of the body would of itself straighten the spine. To keep the spine straight he applied a plaster of paris jacket. "This treatment has proved so successful," says Dr. Foster, "that we are almost justified in expecting that before many years hunchbacks will be unknown." In operating on clubfoot in children, Dr. A. M. Phelps had achieved similar success, and Dr. Adoniram B. Judson won fame in treating curvature of the spine by means of rotation.

Many a child died in former times from croup because the doctor hastened its end by forcing down repulsive medicines. In more recent times, because of the genius of Dr. Horace Green and Dr. Joseph O'Dwyer, both of this city, it was shown that an instrument might be passed through the opening of the larynx Dr. O'Dwyer finally invented a tube which could be thrust into the larynx and left there, so that the patient might breathe through it in case of croup or diphtheria.

The treatment of appendicitis is of American origin, and it is due, according to Dr. Foster, to such men as Dr. Willard Parker, of New York; Dr. Reginald H. Fitz, of Boston; Dr. Theodore A. McGraw, of Detroit; Dr. William S. Halsted, of Baltimore; Dr. John B. Deaver, of Philadelphia, and Dr. Francis H. Markoe, of New York, that the surgery of the abdominal organs has become so daring and yet so successful.*

The specialist is on the increase in this city as in the other densely populated centers of the country; but he is not yet as numerous as the family physician of the old school. In the wealthy districts of the city, one sees the specialist's sign with a striking frequency; but he is found less and less often as one approaches either river. On the East Side the "wholesale doctor" is becoming more and more popular. The "wholesale doctor" is an importation from the continent of Europe. He is employed by mutual benefit societies at a lump sum a year to care for its members. Such organizations are especially numerous in Germany, and they have been bitterly fought by the physicians there, who say the system cheapens their profession, and prohibits a doctor asking large enough fees to repay him for a long and expensive education. In Leipsic a great number of doctors have formed a union in order to boycott any doctor who is employed by a society.

In the country districts one finds the family doctor still, who, like his predecessor of a century ago, treats all kinds of ills under all sorts of conditions. His life is a hard one, and his hours of eating and sleeping are as irregular as those of a fireman. On winter nights he is most likely to be routed out of bed to attend a sick call miles away. What such a man may endure in the dis-

* To this list may be added the names of Mynter, Fowler and Price.—EDITOR.

charge of professional duties was shown by the ordeal of Dr. John Hodge, on the night the second Niagara suspension bridge was wrecked by a hurricane. The doctor lived on the New York shore and was returning from a sick call in Canada, when he was caught in the storm. The bridge swayed so violently that he was nearly thrown into the chasm beneath. His clothes were literally torn from his body by the time he had reached land.

Some 25,000 students are graduated each year from the medical schools of the country, and the majority of them go forth to practice in towns and cities. Less than one-tenth, judging from a recent census of classes recently graduated at the Harvard Medical School, take up work in communities of less than 10,000 inhabitants. Of 487 of these graduates, 191 were practising in cities of over 100,000, 229 in towns of over 10,000 and less than 100,000, and 38 in villages of less than 10,000. Two hundred and twelve were general practitioners, 96 were specialists, and 99 regarded themselves as both; 77 were general practitioners who expected to become specialists. The lines of work which the specialists had picked out were as follows: Surgery,42; gynecology, 19; medicology, 12; otology, 9; laryngology, 19; aurist and oculist, 7; pathology, 7; dermatology, 7; oculist, 6; eye, ear, nose and throat, 5; chemistry, 5; orthopedics, 5; children's diseases, 4; neurology, 4; genitourinary,3; bacteriology, 3; insanity, 3; anatomy, 2; physical training, 2; obstetrics, 2; gastro-intestinal, 1; nutrition, 1; aurist, 1; lungs, 1; dentistry, 1; child, 1; stomach, 1; anesthetist, 1; military medicine, 1; pediatrics, 1; bacteriology, 1; internal medicine and nerves, 1.

